# Digital Support for Family Caregivers: Potential and Challenges of a Hypothetical AI Care Companion

**DOI:** 10.3390/healthcare14050586

**Published:** 2026-02-26

**Authors:** Laura Schwedler, Thomas Ostermann, Jan Ehlers, Gregor Hohenberg

**Affiliations:** 1 Stabsstelle für Digitalisierung und Wissensmanagement, Hochschule Hamm-Lippstadt, 59063 Hamm, Germany; gregor.hohenberg@hshl.de; 2 Fakultät für Gesundheit, Universität Witten-Herdecke, 58455 Witten, Germany; thomas.ostermann@uni-wh.de (T.O.); jan.ehlers@uni-wh.de (J.E.)

**Keywords:** family caregivers, digital support, AI care companion, psychosocial burden, acceptance research, caregiving science

## Abstract

**Background/Objectives**: Family caregivers play a central role in the provision of long-term home-based care and often provide unpaid support over extended periods. This role is associated with substantial psychological, physical, social, and financial burden. Despite high support needs, access to psychosocial services remains limited for many family caregivers. Against this background, AI-based care companions are discussed as a potential low-threshold supplement to existing support structures. The objective of this study was to explore subjectively perceived family caregiver burden and to examine expectations, acceptance conditions, and concerns regarding a hypothetical AI-based care companion, rather than to evaluate effectiveness. **Methods**: An exploratory mixed-methods study was conducted using an anonymous online survey. Perceived family caregiver stress was assessed using self-developed, non-validated ordinal items, including a single-item global burden rating and categorical stress domains. The questionnaire combined closed-ended items (Likert-scale and multiple-choice) with one open-ended question to assess perceived stress, experiences with psychosocial support, and attitudes toward a hypothetical AI care companion. Participants were recruited via an online caregiving course platform. Data collection was voluntary and anonymous and took place in Germany between October and November 2025. Quantitative data were analyzed descriptively and exploratorily, and qualitative responses were analyzed using thematic analysis. **Results**: Fifty-five family caregivers participated in the survey. Overall, perceived family caregiver burden was high, with psychological stress most frequently identified as the dominant stress domain. Difficulties in accessing psychosocial support were reported by 58% of the respondents. Willingness to consider using an AI-based care companion varied by degree of acceptance: 36% reported clear willingness, 31% expressed conditional or tentative willingness, and 33% indicated reluctance or rejection. The most frequently selected expected functions included emotional support, early detection of overload, and caregiving-related information. Data protection, professional reliability, and concerns regarding incorrect advice were identified as the most relevant perceived risks. **Conclusions**: The findings reflect family caregivers’ perceived burden and anticipated needs, highlighting persistent gaps in psychosocial support. From the perspective of respondents, a hypothetical AI-based care companion could represent a complementary support option if it provides personalized, non-judgmental, and reliable assistance. These results describe perceived potential and acceptance conditions, not verified efficacy. Further research, including prototype development, usability testing, and pilot studies, is required to examine feasibility, ethical implications, and real-world impact.

## 1. Introduction

Family caregivers play a central role in the provision of long-term care, particularly in home-based settings. In Germany, the majority of people in need of care are supported by relatives who provide unpaid and often long-term informal care, frequently alongside employment and other family responsibilities [1,2,3,4]. This form of care is indispensable for the sustainability of the health and long-term care system, yet it is associated with substantial multidimensional burden for those providing care.

A large body of research demonstrates that caregiving is associated with psychological, physical, social, and financial stress [5,6,7,8,9,10,11,12]. Psychological stressors include emotional overload, chronic worry, anxiety, and feelings of being overwhelmed, while social stress often manifests as isolation, role conflicts, or reduced participation in social life. At the same time, caregiving involves significant physical demands, such as lifting, mobilizing, repetitive movements, disturbed sleep, and prolonged fatigue, which may negatively affect musculoskeletal health and overall physical well-being [4,7,13]. From a human movement science perspective, these physical demands are characterized by repetitive movement patterns, prolonged static postures, and insufficient recovery periods, which are closely linked to fatigue, musculoskeletal strain, and functional limitations. Importantly, these dimensions interact dynamically: physical exhaustion may intensify psychological distress, while persistent emotional strain can reduce physical resilience and recovery capacity [5,8,14].

This burden is particularly pronounced in family caregivers of people with chronic and progressive conditions such as dementia, where caregiving often extends over many years and involves high emotional involvement, continuous vigilance, and increasing care complexity [6,7,8]. Recent epidemiological data show that caregiving demands are rising further. In Germany, average weekly caregiving time increased from 43 h in 2019 to 49 h in 2023, with many family caregivers reducing working hours or leaving employment altogether [3,4,15]. These developments have major implications not only for family caregivers’ health and quality of life but also for the sustainability of the health and social care system.

Despite high levels of burden, access to psychosocial and therapeutic support remains limited for many family caregivers. Long waiting times, bureaucratic barriers, financial constraints, lack of information, time scarcity, and feelings of shame or self-stigmatization often prevent family caregivers from using existing services [9,10,13,15,16]. These unmet psychosocial needs represent a persistent gap in outpatient care.

Digital health solutions have increasingly been discussed as a means of alleviating family caregiver burden. Existing applications primarily focus on organizational and practical aspects such as medication management, appointment coordination, or documentation. While these tools address specific caregiving tasks, they rarely target emotional support, coping strategies, or individualized psychosocial counseling in an integrated manner [10,11,12,16,17,18]. Thus, a gap exists not only in psychosocial care provision itself, but also in current digital interventions that insufficiently address family caregivers’ emotional and subjective burden.

Against this background, artificial intelligence (AI)-based support systems have been proposed as a potential extension of digital health services. AI-supported applications, such as conversational agents or digital companions, could theoretically offer low-threshold, continuous, and personalized support. Possible roles include providing evidence-based information, supporting self-reflection and coping strategies, monitoring stress-related indicators through self-reported data, and facilitating early identification of overload. Importantly, such systems are not intended to replace professional care but to complement existing support structures and facilitate access to human counseling when needed [19,20,21,22,23].

However, the application of AI in caregiving contexts also raises critical questions. Concerns regarding data protection, transparency, professional reliability, emotional appropriateness, and overreliance on technology are widely discussed in the literature [20,22,24,25,26]. Empirical evidence regarding family caregivers’ acceptance, expectations, and perceived risks of AI-based support remains limited, particularly within the German care context, which is characterized by a strongly regulated health system, specific reimbursement structures, and legal frameworks for counseling and prevention services.

Furthermore, family caregivers’ attitudes toward digital support are likely shaped by contextual factors such as caregiving duration, intensity of care, and type of caregiving tasks. These factors influence both perceived burden and openness toward technological assistance. In particular, higher psychological burden may function as a motivational driver for seeking low-threshold digital support, whereas physical workload and time constraints may affect patterns of use and expectations of such systems. A clearer understanding of these relationships is essential for needs-oriented and ethically responsible system design. These relationships are examined in the present study in an exploratory and descriptive manner and are not interpreted as causal effects but as contextual patterns informing needs-oriented system design.

Against this empirical and conceptual background, the development of a hypothetical model of an “AI care companion” serves as a useful analytical framework. This model does not aim to evaluate an existing intervention or demonstrate effectiveness but rather to explore family caregivers’ perceived needs, expectations, acceptance conditions, and concerns regarding AI-supported assistance. Such an exploratory approach is a necessary prerequisite for user-centered development of future digital support solutions.

Therefore, the aim of this study is to present an exploratory analysis of family caregivers’ stress experiences and their attitudes toward a hypothetical AI care companion. Specifically, this study examines: (1) perceived levels and dominant dimensions of family caregiver burden, (2) experiences with and barriers to psychosocial support, (3) expectations, acceptance conditions, and concerns related to AI-based support, and (4) the relationship between perceived burden and willingness to use such systems. By systematically linking subjective burden, unmet psychosocial needs, and acceptance of AI-based assistance, this study contributes to the early conceptualization of AI-supported digital care companions for family caregivers. The findings describe perceived potential and anticipated requirements rather than verified effectiveness and provide a needs-oriented basis for future prototype development, usability testing, and pilot studies.

## 2. Materials and Methods

### 2.1. Study Design

This study followed an exploratory mixed-methods design using an anonymous online survey. Quantitative items (Likert-scale and multiple-choice questions) were combined with one open-ended qualitative question to assess perceived family caregiver burden, experiences with psychosocial support, and acceptance, expectations, and concerns regarding a hypothetical AI-based care companion.

Given the absence of established instruments specifically addressing the acceptance and perceived usefulness of AI companions for family caregivers, the study was designed as hypothesis-generating and descriptive. The qualitative component consisted of a single open-ended question and was not intended to generate in-depth qualitative theory or achieve data saturation. Instead, it served a contextualizing function by capturing participants’ own perspectives and language to complement and enrich the quantitative findings.

The study aimed to explore subjective perceptions and anticipated requirements rather than to evaluate clinical effectiveness or actual usage behavior of an AI system.

### 2.2. Recruitment, Procedure, and Ethical Considerations

Participants were recruited via an online nursing and caregiving course platform addressing family caregivers. The study was introduced as a voluntary survey on family caregiver burden and digital support options. Data collection took place in Germany between October and November 2025.

Participation was voluntary and anonymous, and no financial incentives were provided. Before starting the survey, participants received written study information and provided electronic informed consent. No personally identifiable data were collected.

The study was approved by the Ethics Committee of Witten/Herdecke University (No. 184/2023, 22 August 2023) and conducted in accordance with the Declaration of Helsinki.

Sample size was determined by feasibility within the recruitment platform and intended as exploratory rather than confirmatory. The study was designed to identify patterns of perceived burden and acceptance of AI-based support rather than to estimate prevalence or test hypotheses. Recruitment via an online platform likely favored family caregivers with higher digital literacy, which may have influenced acceptance of digital solutions.

### 2.3. Survey Instrument and Operationalization

The questionnaire consisted of 17 self-developed items based on established theoretical models and current empirical literature on family caregiver burden, psychosocial stress, digital health, and technology acceptance [1,2,3,4,5,6,7,8,9,10,11,12,13,14,15,16,18,19,20,21,22,24,25,26,27,28,29,30,31,32,33,34]. No standardized or validated family caregiver burden scales were applied.

This approach was chosen to minimize respondent burden, accommodate heterogeneous caregiving situations, and allow flexible exploration of attitudes toward a hypothetical AI-based support system. However, the use of non-validated instruments limits measurement reliability and comparability with existing studies using established scales such as the Zarit Burden Interview (ZBI) or the BSFC-s.

The questionnaire comprised 16 quantitative items and one open-ended qualitative question. For transparency and reproducibility, the full questionnaire is provided as Supplementary Material.

#### 2.3.1. Perceived Stress of Family Caregivers

Perceived stress was assessed using a single-item global self-report measure capturing participants’ overall subjective appraisal of caregiving-related burden [5,6,7,14,15,27,28,35].

Participants were asked:

“How strongly do you feel burdened overall by your caregiving activities?”

Responses were recorded on a 5-point Likert scale ranging from not at all burdened to very strongly burdened.

Single-item global burden measures have been used in family caregiver and stress research as parsimonious screening tools when respondent burden must be minimized and when heterogeneous caregiving situations are assessed. This approach captures subjective appraisal rather than clinical symptom severity and is suitable for exploratory needs assessments. Previous research has shown that single-item family caregiver burden ratings are moderately to strongly correlated with established multidimensional burden scales and can provide valid approximations of subjective overall burden in exploratory or screening contexts, particularly when questionnaire length and respondent burden must be kept low.

However, this measure does not allow multidimensional differentiation of psychological, physical, and social burden and limits comparability with validated family caregiver burden instruments. The results, therefore, reflect perceived overall strain rather than clinically defined burden.

#### 2.3.2. Stress Domains

To contextualize overall stress, participants were asked to identify one dominant stress domain perceived as most burdensome. The following categories were provided:

- Psychological/emotional stress;
- Physical stress;
- Social stress;
- Financial stress.

These domains were derived from established caregiving stress literature [1,2,4,5,6,7,8,9,10,12,13,14,15,27,35].

Physical workload and movement-related strain were assessed only at the level of perceived dominant burden domain and not in terms of intensity, frequency, or functional impairment. This categorical approach was chosen to differentiate perceived stress patterns rather than to quantify physical load or biomechanical exposure. Consequently, conclusions regarding physical strain remain descriptive and cannot be interpreted as measures of functional limitation or health outcomes.

Only the dominant stress domain was assessed rather than the simultaneous presence of multiple burden dimensions. This forced-choice approach simplifies complex and co-occurring burdens and may bias responses toward more salient or easily articulated psychological stress.

#### 2.3.3. Experiences with Psychological/Psychosocial Support

Experiences with psychosocial support were assessed using two multiple-choice items adapted from prior family caregiver research [7,10,11,15,16,21,29,30]:

- Prior difficulties in accessing support (yes/no/not attempted);
- Perceived barriers (e.g., waiting times, costs, lack of time, shame, lack of information).

#### 2.3.4. Acceptance of a Hypothetical AI Care Companion

Acceptance-related constructs were informed by the Technology Acceptance Model (TAM), UTAUT2, and research on AI-based health applications [18,19,20,22,23,24,25,26,31,32,33]. The following aspects were assessed using Likert-scale items:

- General willingness to use an AI care companion;
- Importance of non-judgmental communication;
- Importance of personalization;
- Expected frequency of use.

Acceptance and willingness to use were assessed with reference to a hypothetical AI-based care companion. As no prototype was presented, responses reflect anticipated attitudes and perceived usefulness rather than actual usage behavior, feasibility, or clinical implementation.

#### 2.3.5. Expected Functions, Devices, and Concerns

Participants selected preferred functions (e.g., emotional support, early overload detection, information), preferred devices (smartphone, tablet, computer, voice assistant), and perceived concerns (e.g., misinformation, data protection, lack of empathy) based on prior research on digital health applications and AI companions [10,11,12,16,20,21,22,23,24,25,26,30].

#### 2.3.6. Sociodemographic and Caregiving Characteristics

The following variables were assessed:

- Age;
- Gender;
- Educational level;
- Duration of caregiving;
- Average weekly caregiving hours.

The relationship between family caregiver and care recipient (e.g., spouse, adult child) and specific diagnoses (e.g., dementia) were not assessed and represent a substantial limitation, as burden profiles differ markedly across caregiving relationships and disease characteristics.

#### 2.3.7. Literature Base and Criteria for Instrument Development

The questionnaire was developed based on a systematic literature review conducted in the following databases:

- PubMed;
- CINAHL;
- PsycINFO;
- Web of Science;
- Google Scholar;
- Nursing and health science portals;

Search terms included combinations of:

- Informal caregiver, family caregiver, caregiver burden, caregiver stress, dementia caregiver;
- Psychological distress, emotional burden, fatigue, sleep quality, coping;
- Digital health, AI companion, AI chatbot, telemedicine, digital therapeutics, DiGA, DiPA;
- Technology acceptance, UTAUT, TAM, trust in AI.

The search period covered publications from 2020 to 2025. Studies in German and English were considered.

Inclusion criteria:

- Empirical studies, systematic reviews, meta-analyses;
- Focus on family caregivers;
- Outpatient/home care settings;
- Peer-reviewed or scientifically recognized publications.

Exclusion criteria:

- Purely technical AI development without user focus;
- Studies exclusively on professional caregivers;
- Inpatient-only settings;
- Non-scientific publications.

The literature review informed both the conceptual structure and the content of the questionnaire items. Item development was guided by three main theoretical strands:

1. Multidimensional family caregiver burden and stress models, encompassing psychological, physical, social, and financial burden;
2. Technology acceptance frameworks, particularly the Technology Acceptance Model (TAM) and UTAUT2, addressing perceived usefulness, ease of use, and behavioral intention;
3. Empirical research on digital and AI-based support systems for family caregivers, focusing on expectations, perceived benefits, and concerns such as trust, data protection, and emotional appropriateness.

Item formulation followed predefined criteria:

(a) Conceptual relevance to family caregiver burden or AI acceptance;
(b) Clarity and comprehensibility for non-professional caregivers;
(c) Minimal respondent burden;
(d) Suitability for exploratory needs assessment rather than clinical diagnosis.

Given the exploratory nature of the study and the absence of validated instruments specifically addressing acceptance and expectations of AI-based care companions for family caregivers, no standardized scale could be directly applied. Established family caregiver burden instruments (e.g., Zarit Burden Interview, BSFC-s) were not included in order to reduce questionnaire length and respondent burden and to allow flexible coverage of heterogeneous caregiving situations.

This methodological decision represents a trade-off between psychometric precision and feasibility. While the use of self-developed items limits comparability with existing family caregiver burden research, it enabled a first needs-oriented exploration of perceived burden and anticipated requirements for an AI-based support system.

The complete questionnaire and its theoretical allocation are provided as Supplementary Material to ensure transparency and reproducibility.

### 2.4. Validation Procedures

#### 2.4.1. Expert Validation

Content validity was assessed by a multidisciplinary expert panel (n = 3) from nursing science and digital health with professional experience ranging from 6 to 20 years. Experts independently evaluated items for relevance, clarity, theoretical alignment, and practical applicability. Discrepancies were discussed until consensus was reached.

Revisions included rewording items addressing AI-related risks, improving differentiation between acceptance and concerns, and simplifying stress-related wording to reduce ambiguity.

Inter-rater agreement was achieved through consensus discussions rather than statistical indices.

#### 2.4.2. Pretest with Family Caregivers

A cognitive pretest was conducted with eight family caregivers recruited through community networks. Participants represented varying age groups (ranging from early adulthood to older adulthood), different caregiving durations (from less than one year to more than ten years), and diverse caregiving contexts, including care for partners, parents, and other relatives. This heterogeneity was intended to capture variation in caregiving experience and comprehension patterns. Using think-aloud elements, comprehensibility, emotional burden, and response effort were assessed.

Based on feedback, item wording was shortened, explanatory examples were added, and the distinction between subjective stress perception and clinical diagnosis was clarified. Open-ended questions were simplified to reduce emotional and cognitive burden.

### 2.5. Data Analysis

In line with the exploratory mixed-methods design, qualitative findings were used to contextualize and deepen the interpretation of quantitative patterns rather than to triangulate or validate them.

#### 2.5.1. Quantitative Analysis

Quantitative data were analyzed using IBM SPSS Statistics Version 29 (IBM Corp., Armonk, NY, USA). Descriptive statistics were calculated for all items.

Exploratory analyses included:

- Spearman correlations between perceived stress and willingness to use AI;
- Kruskal–Wallis tests for group differences (age, gender, caregiving duration);
- Chi-square tests for categorical associations.

These analyses were exploratory and intended to identify patterns rather than test hypotheses.

#### 2.5.2. Qualitative Analysis

The qualitative component consisted of a single open-ended question addressing expectations and requirements for a helpful AI-based care companion. Responses were analyzed using a descriptive thematic approach. Short text responses were reviewed iteratively, and recurring content patterns were summarized into overarching thematic categories.

The qualitative analysis did not aim to achieve theoretical saturation but served to contextualize and enrich the quantitative findings.

### 2.6. Methodological Limitations

Due to the exploratory design, self-developed non-validated instruments, small sample size, and online recruitment strategy, findings are not generalizable and reflect perceived burden and anticipated usefulness rather than objective stress levels or intervention effectiveness.

The reliance on a single-item global stress measure and a forced-choice dominant stress domain simplifies complex, co-occurring burdens and limits multidimensional interpretation. The absence of validated burden scales restricts comparability with existing family caregiver research.

Recruitment via an online platform likely favored digitally literate family caregivers and may have biased acceptance of AI-based support. family Caregiver-care recipient relationships and specific diagnoses were not assessed, limiting interpretive depth.

Finally, acceptance was assessed for a hypothetical AI-based care companion without a prototype; therefore, results reflect anticipated attitudes rather than actual usage behavior or feasibility.

## 3. Results

### 3.1. Sample

A total of 55 family caregivers participated in the online survey. The sample showed considerable heterogeneity with regard to caregiving duration and weekly caregiving hours. While some participants had been providing care for less than six months and spent fewer than ten hours per week on caregiving, others reported caregiving periods of more than five years and weekly caregiving times exceeding 30 h.

Due to the relatively small sample size and the heterogeneity of caregiving situations, the results should be interpreted as exploratory and cannot be assumed to represent the full variability of caregiving burden among family caregivers in Germany.

The detailed sociodemographic characteristics are presented in Table 1.

To facilitate interpretation, sociodemographic characteristics are reported as absolute numbers and percentages.

### 3.2. Perceived Stress (Quantitative Results)

**Figure 1:**
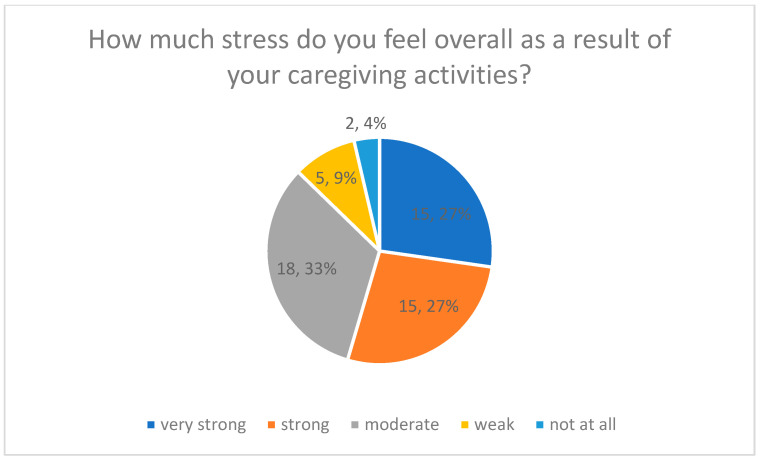
Overall Stress from Caregiving Activities.

Overall caregiving-related stress was assessed using a single-item self-report measure on a 5-point Likert scale ranging from 1 = not at all stressful to 5 = very stressful (Figure 1). All results presented below should be interpreted as descriptive and exploratory, reflecting subjective perceptions within a small and heterogeneous sample rather than comprehensive assessments of family caregiver burden.

The results indicate a high perceived burden among participants. In total, 27% rated their caregiving situation as very strong (Likert value 5), and another 27% as strong (Likert value 4). Approximately 30% reported a moderate level of stress (Likert value 3), while 13% indicated weak or no stress (Likert values 1–2).

Figure 1 displays the distribution of perceived overall stress levels, with percentage values shown for each response category.

#### Particularly Stressful Areas (Categorical Quantitative Results)

To further contextualize perceived stress, participants were asked to indicate one dominant stress domain they perceived as most burdensome. This item was assessed as a categorical multiple-choice question. Participants were asked to select one dominant stress domain only. This forced-choice format does not reflect the simultaneous presence of multiple burden dimensions and therefore captures perceived priority rather than overall burden profiles.

**Figure 2:**
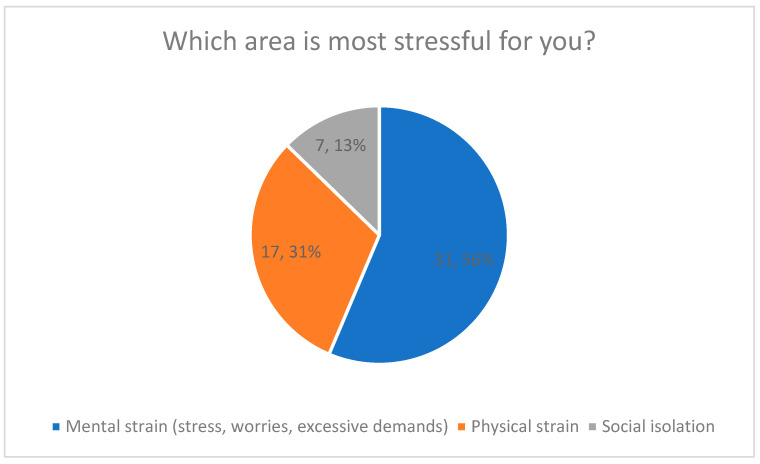
Most Stressful Areas of Caregiving.

More than half of respondents (56%) identified psychological stress (e.g., emotional strain, worries, feelings of being overwhelmed) as their primary burden (Figure 2). Physical stress was selected by 31%, while 13% reported social isolation as their most burdensome stressor.

These domains represent distinct stress categories rather than combined dimensions. No composite stress score was calculated.

Figure 2 presents the relative frequencies of the reported dominant stress domains, including percentage values for each category.

### 3.3. Difficulties in Accessing Support (Quantitative Results)

**Figure 3:**
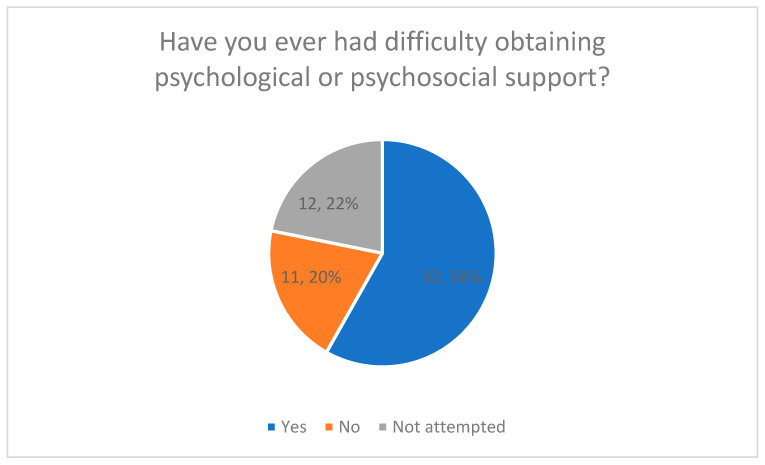
Difficulties in Obtaining Psychological or Psychosocial Support.

Difficulties in accessing psychological or psychosocial support were assessed using a closed multiple-choice question (Figure 3).

Overall, 58% of respondents reported having experienced difficulties in accessing such support. In total, 20% reported no difficulties, while 22% indicated that they had not yet attempted to seek psychological or psychosocial support.

Percentage values are displayed directly in Figure 3.

#### Perceived Barriers (Quantitative Results)

Participants who reported difficulties were asked to indicate perceived barriers using a multiple-response item. The most frequently reported barrier was long waiting times (45%), followed by financial or cost-related barriers (29%). Shame or personal inhibitions were reported by 16%, while 10% selected no or other barriers.

**Figure 4:**
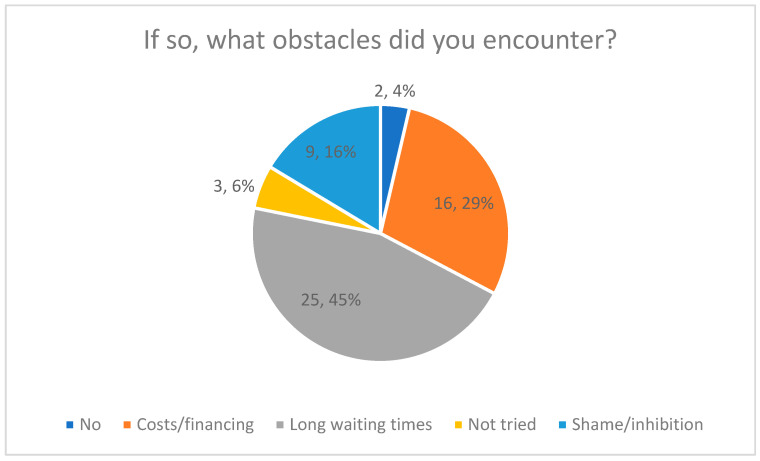
Encountered Obstacles in Using a Digital AI Care Companion.

The distribution of barriers is shown in Figure 4, including percentage labels.

### 3.4. Interest in a Digital AI Care Companion (Quantitative Results)

**Figure 5:**
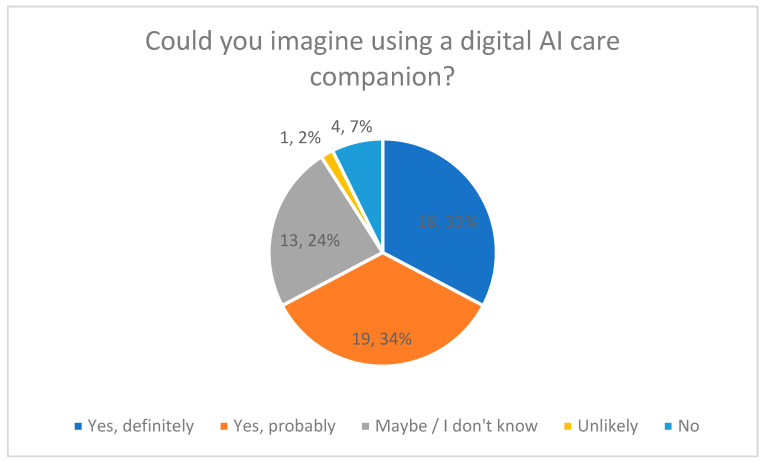
Imagining the Use of a Digital AI Care Companion.

Interest in using a digital AI-based care companion was assessed using a Likert-scale item (Figure 5).

Overall, 67% of respondents indicated that they could imagine using such a tool in their caregiving routine (33% “definitely”, 34% “probably”). A total of 9% stated that they would not consider using an AI-based care companion.

Response distributions with percentage values are shown in Figure 5.

#### 3.4.1. Desired Functions (Quantitative Results)

**Figure 6:**
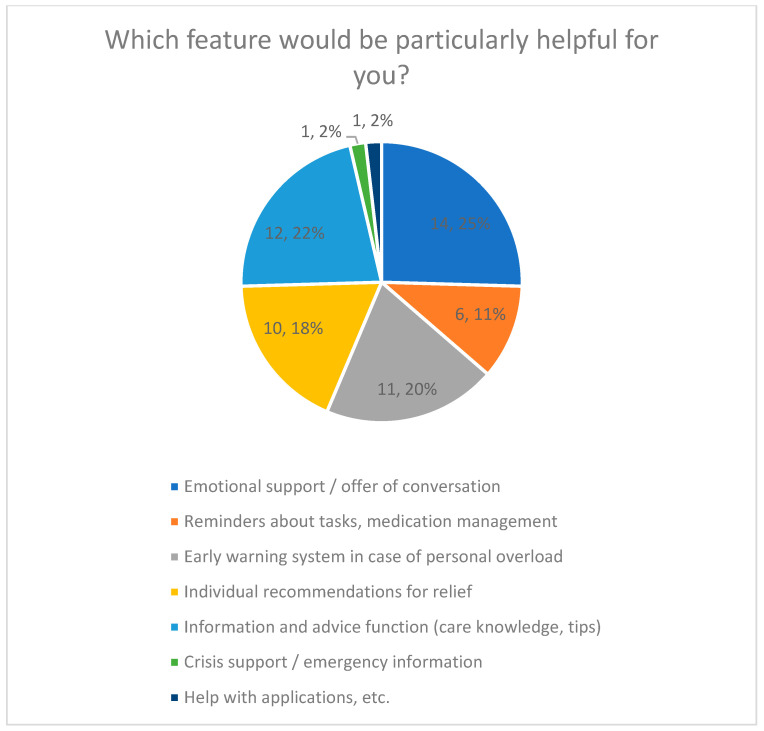
Particularly Helpful Features of an AI Companion.

Desired functions of a hypothetical AI care companion were assessed using a closed multiple-choice question with predefined response options (Figure 6).

Emotional support was the most frequently selected function (25%), although differences between several functions were small (22%). Early detection of overload was chosen by 20%, while individual respite recommendations were selected by 18%.

Less frequently selected functions included medication management and reminders (11%), as well as emergency information and assistance with applications, each selected by 2% of respondents.

The distribution of selected functions is shown in Figure 6, including percentage values for each category.

#### 3.4.2. Preferred Communication Style and Degree of Personalization (Quantitative Results)

**Figure 7:**
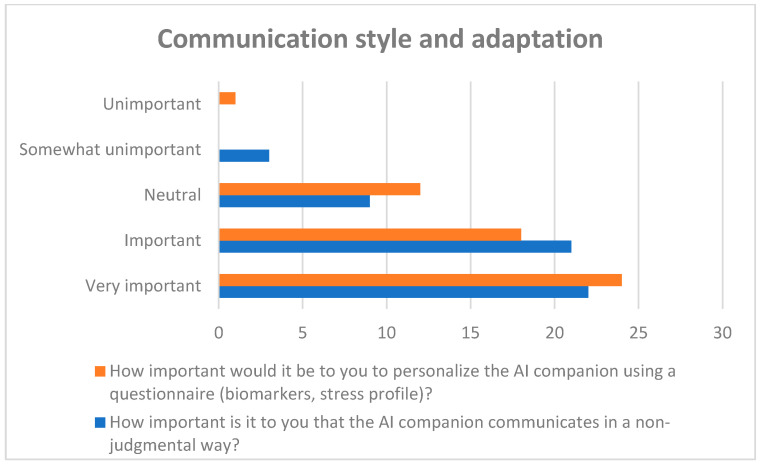
Preferred communication style and degree of personalization.

Preferences regarding communication style and personalization were assessed using Likert-scale items (Figure 7).

A total of 78% of respondents rated non-judgmental communication as important or very important. Regarding personalization, 77% indicated that individual adaptation of the AI companion (e.g., based on personal situation or stress profile) was important or very important.

Response distributions for both items are presented in Figure 7.

#### 3.4.3. Expected Frequency of Use (Quantitative Results)

**Figure 8:**
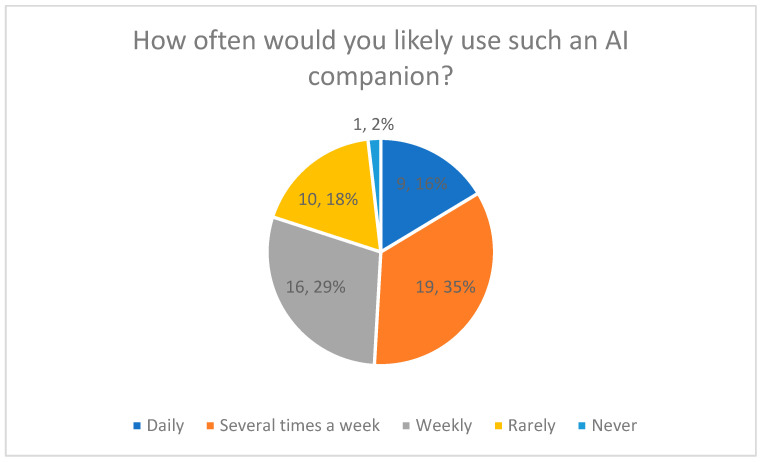
Likely Frequency of Using an AI Companion.

Expected frequency of use of the AI care companion was assessed using a Likert-type frequency item (Figure 8).

Overall, 16% of respondents reported that they would use the AI companion daily, while 35% indicated several times per week. A total of 29% stated that they would use it weekly. The remaining 20% reported that they would use the AI companion rarely or not at all.

The distribution of responses is displayed in Figure 8, including percentage values for each category.

#### 3.4.4. Preferred Devices (Quantitative Results)

**Figure 9:**
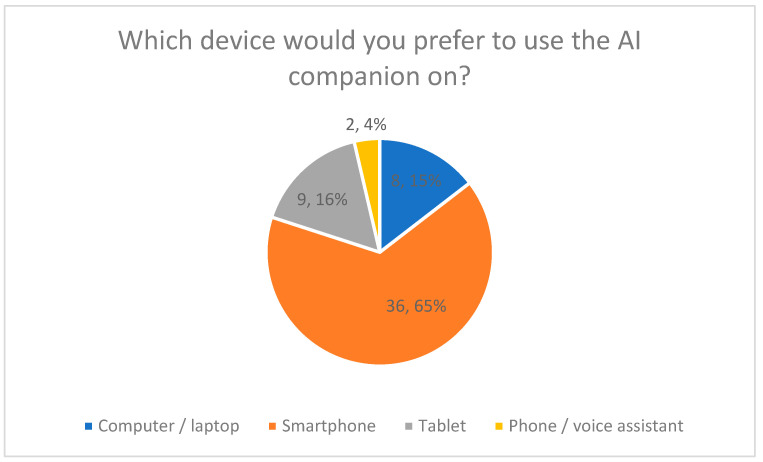
Preferred Device for Using the AI Companion.

Preferred access devices were assessed using a single-choice multiple-choice question (Figure 9).

The majority of respondents (65%) indicated a smartphone as their preferred device for using the AI companion. Tablets were selected by 16%, and computers or laptops by 15%. Voice-based assistants were selected by 4% of respondents.

Percentage distributions are shown in Figure 9.

#### 3.4.5. Perceived Concerns (Quantitative Results)

**Figure 10:**
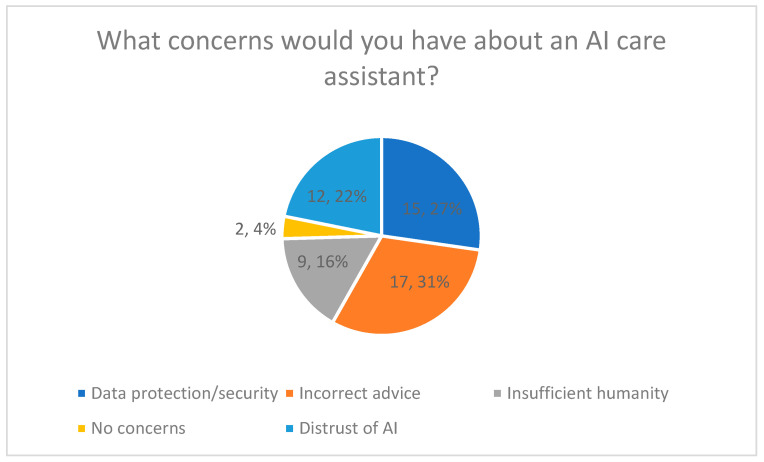
Concerns About an AI Care Assistant.

Perceived concerns regarding the use of an AI-based care companion were assessed using a multiple-choice question allowing multiple responses (Figure 10).

The most frequently reported concern was possible misadvice or lack of professional reliability (31%). Data protection and data security concerns were reported by 27% of respondents. General distrust of AI systems was selected by 22%, while concerns about insufficient emotional quality or empathy were reported by 16%. A total of 4% of respondents reported no concerns.

The distribution of concerns is presented in Figure 10, including percentage values.

#### 3.4.6. Expectations of the AI Companion (Qualitative Results)

Expectations toward the AI-based care companion were assessed using an open-ended question. Responses were analyzed using qualitative content analysis. The responses were short and heterogeneous in length. Analysis followed a descriptive thematic approach, focusing on recurring content patterns rather than interpretative depth.

**Figure 11:**
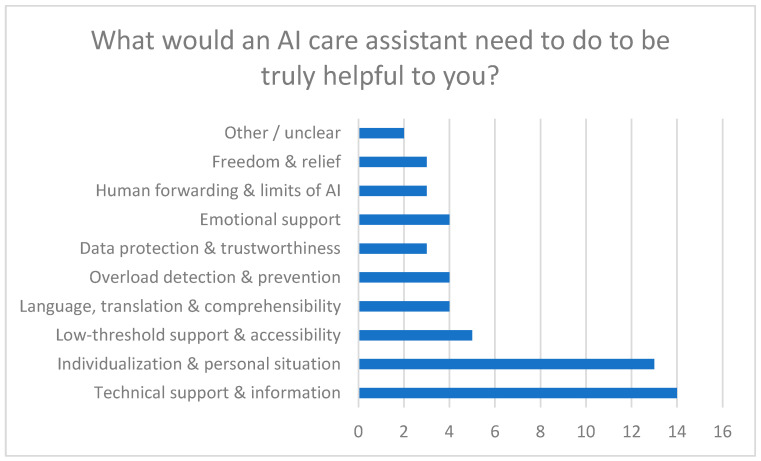
Requirements for a Helpful AI Care Assistant.

Four main expectation areas emerged (Figure 11):

1. Individual and context-sensitive support
  ○ Quote from a participant: “He should advise me individually—not according to a standard pattern.”
2. Low-threshold availability
  ○ Quote from a participant: “My questions should be answered quickly and directly.”
3. Technically accurate and up-to-date information
  ○ Quote from a participant: “They should provide up-to-date information and be familiar with regional support services for relatives.”
4. Connection to human experts
  ○ Quote from a participant: “If you have further questions on the topic or feel that the AI has not understood you correctly, you should have the option of being transferred to a human employee.”

Figure 11 summarizes the qualitative categories derived from the open-ended responses.

### 3.5. Exploratory Quantitative Analyses (Inferential Results)

The following analyses are exploratory in nature and aim to identify potential patterns and associations within the data rather than to test hypotheses or draw generalizable conclusions. Quantitative data were statistically analyzed using IBM SPSS Statistics Version 29 (IBM Corp., Armonk, NY, USA). Descriptive statistics were calculated for all 17 questionnaire items. Mean values ranged from M = 1.22 to M = 4.00, indicating a broad distribution of response tendencies across items addressing family caregiver stress, barriers to support, and acceptance of AI-based assistance. Standard deviations ranged from SD = 0.42 to SD = 2.75, reflecting substantial interindividual variability, particularly regarding the perceived usefulness of AI functions and expected frequency of use.

Given the small sample size, all inferential analyses are characterized by low statistical power. Accordingly, statistical significance should be interpreted with caution, and results are intended to indicate potential patterns rather than robust group differences or predictive relationships.

Reliability analysis

A reliability analysis was conducted for the summarized items assessing attitudes toward AI-based support and evaluation of AI functions. The scale demonstrated very high internal consistency, with a Cronbach’s alpha of 0.983, indicating a high degree of homogeneity among the included items. The very high internal consistency may partly reflect conceptual overlap among acceptance-related items.

Inferential analyses were conducted to explore possible relationships between perceived burden and acceptance-related variables.

Correlation analysis

To examine the association between overall perceived stress and willingness to use an AI-based care companion, a Spearman’s rank correlation was performed. The analysis revealed a strong positive correlation (Spearman’s ρ = 0.92), indicating a monotonic association between higher stress ratings and higher willingness to use AI-based support. The exceptionally high correlation may be influenced by common method variance, the use of single-item self-report measures, and the hypothetical nature of the AI acceptance item. Therefore, this association should not be interpreted as evidence of a causal relationship.

Group comparisons

Group differences in willingness to use an AI-based care companion were examined using Kruskal–Wallis tests for age groups, gender, and duration of caregiving. The analysis yielded a statistically significant result (H = 65.57, *p* < 0.00001), indicating that at least one group differed from the others with regard to willingness to use AI-based support. Post hoc comparisons were not conducted due to limited sample size and exploratory intent.

Chi-square analysis

A chi-square test was conducted to examine the association between categorized stress levels and categorized willingness to use AI. The test did not indicate a statistically significant association (χ^2^ = 1.23, *p* = 0.268; Yates-corrected: χ^2^ = 0.79, *p* = 0.376).

Given the small sample size, low statistical power, and reliance on self-reported single-item measures, these inferential results should be interpreted cautiously and viewed as indicative trends rather than robust statistical evidence.

Confidence intervals were not calculated due to the exploratory nature of the study and the limited sample size. The analyses focus on identifying directional trends rather than estimating population parameters.

Overall, inferential findings are reported to highlight potential relationships that warrant further investigation in larger, theory-driven studies.

## 4. Discussion

### 4.1. Interpretation of the Stress Situation of Family Caregivers

The findings of the present study indicate a high overall subjective burden among family caregivers, with psychological strain being reported more frequently as the dominant stressor than physical or social burden. This pattern is consistent with previous research highlighting emotional stress, constant responsibility, and perceived overload as central components of family caregiver burden [1,2,3,4,7,9,14,15]. However, this result must be interpreted cautiously and in close relation to the applied measurement approach.

Burden in the present study was assessed exclusively using self-reported indicators, which are particularly sensitive to subjective emotional strain, perceived stress, and mental exhaustion [27,28,35]. Consequently, the predominance of psychological stress observed here should not be interpreted as evidence of a lower relevance of physical demands. Rather, physical strain and fatigue may be underrepresented because they are frequently experienced and articulated through psychological symptoms such as exhaustion, sleep disturbances, or emotional depletion. Previous studies demonstrate strong interdependencies between physical workload, chronic fatigue, and psychological stress in caregiving contexts, suggesting that these dimensions cannot be meaningfully separated on the basis of self-report alone [13,14,28,35].

In addition, the identification of a single dominant stressor was based on a forced-choice item, requiring participants to select only one stress domain. This format captures perceived priority rather than the simultaneous presence of multiple burden dimensions and therefore constrains interpretation. The reported stress patterns should thus be understood as indicative of what family caregivers experience as most salient, rather than as comprehensive representations of their overall burden profiles.

Exploratory correlation analyses revealed a strong positive association between perceived overall stress and willingness to use an AI-based care companion. While this finding suggests that family caregivers experiencing higher subjective burden may be more open to digital support, it should not be interpreted as reflecting a stable or general attitude toward AI technologies. Instead, the observed association likely represents situational and context-dependent openness driven by current stress levels. The exceptionally high Spearman correlation coefficient (ρ = 0.92) must be interpreted with particular caution, as it may be influenced by sample homogeneity, ordinal scaling effects, common method variance, and the use of single-item self-report measures. No causal conclusions can be drawn from this association.

Overall, the stress patterns identified in this study align with existing national and international research on family caregiving [5,6,7,13]. At the same time, the results remain exploratory and cannot be generalized to the broader population of family caregivers. They should be interpreted as reflecting perceived burden and needs within a small, heterogeneous sample rather than as prevalence estimates or comprehensive assessments of family caregiver stress.

### 4.2. Potential of an AI-Based Care Companion

Against this background, the present study provides exploratory insights into how an AI-based care companion might address gaps perceived by family caregivers. Importantly, these conclusions are derived from the alignment between reported stress patterns and functions rated as helpful by respondents, rather than from an evaluation of an existing intervention or evidence of effectiveness.

The most frequently desired functions—emotional support, early detection of overload, individualized recommendations, and reliable caregiving-related information—correspond to areas in which family caregivers reported high strain and limited access to timely support. Similar functional expectations have been described in previous research on digital health interventions for family caregivers, particularly regarding emotional support, information provision, and personalization [10,11,12,16,21,22,33]. The present findings add empirical support to the relevance of these functions from the perspective of family caregivers themselves, while remaining limited to expressed preferences and expectations.

The observed monotonic relationship between perceived stress and willingness to use an AI-based care companion further suggests that openness toward digital support may increase under conditions of higher subjective burden. Notably, categorical analyses did not reveal statistically significant associations, indicating that gradients in stress perception may be more accurately captured through ordinal analyses. This discrepancy underscores the exploratory nature of the findings and cautions against overinterpretation.

While emotional relief emerged as the most prominent expectation, the results also raise hypotheses regarding potential indirect effects on physical workload and fatigue. Functions such as early warning systems, reminders, and individualized respite recommendations could theoretically be perceived as supporting self-management, pacing of caregiving tasks, and prevention of overload. However, as no direct measures of physical workload, movement characteristics, or bodily strain were collected, these considerations remain hypothetical. Any association between AI-based support and physical burden should therefore be interpreted as a conceptual implication derived from participant expectations rather than as empirical evidence.

The strong emphasis on personalization aligns with participatory design research, indicating that family caregivers prefer adaptive, context-sensitive systems over generic solutions [17,23,33]. Nonetheless, the present data do not allow conclusions about actual usability, effectiveness, or long-term engagement. The findings should be understood as outlining acceptance conditions rather than demonstrating benefits.

Exploratory group comparisons suggested differences in willingness to use AI-based support across age groups and caregiving duration. Although statistically significant, these results are characterized by low statistical power and should be interpreted cautiously. They point toward the potential relevance of life-course and contextual factors for acceptance but require confirmation in larger, theory-driven studies.

### 4.3. Limitations, Risks, and Challenges

Several limitations must be considered when interpreting the present findings. First, the small sample size and cross-sectional design limit generalizability and preclude causal inference. All analyses were exploratory in nature and intended to identify potential patterns rather than to test hypotheses or estimate population parameters.

Second, reliance on self-reported, predominantly single-item measures restricts differentiation between psychological, physical, and social dimensions of burden and may have contributed to the observed dominance of psychological stress. Objective measures of workload, movement, or physiological strain were not included and should be incorporated in future research.

Third, recruitment via an online survey may have introduced self-selection bias toward digitally literate family caregivers, potentially inflating acceptance of AI-based support. This raises concerns about digital inequality: family caregivers with high physical workload, limited technical access, or lower digital literacy may be underrepresented and could be disproportionately excluded from digital support solutions. Without careful design and implementation, AI-based tools risk reinforcing existing disparities rather than alleviating them.

Ethical challenges also warrant attention. Concerns regarding data protection, privacy, and incorrect advice reflect broader debates on digital health ethics and regulation [19,22,23,24,25,31,33]. In addition, while AI companions may offer perceived emotional relief, there is a risk of overreliance or unintended substitution of human relationships. From an ethical perspective, this underscores the importance of designing AI-based support in a way that preserves family caregiver autonomy and avoids dependency or unintended replacement of human care relationships. These issues highlight the need for transparent system boundaries, explainability, and clear escalation pathways to human professionals.

### 4.4. Financing, Implementation, and Care Context

From a structural perspective, the findings must be situated within a care system that currently lacks dedicated reimbursement pathways for digital tools targeting family caregivers. Participants’ reported barriers, particularly cost-related obstacles and long waiting times, underscore the importance of low-threshold, publicly supported implementation models.

Potential entry points include integration into counseling and support services under SGB XI, preventive and health promotion programs under SGB V, or evaluation within model projects and pilot studies [24,25,31]. Embedding AI-based systems into established care counseling services (e.g., §7a SGB XI) or municipal care networks could help ensure linkage to human expertise and local support structures, mitigating risks associated with autonomous digital guidance.

The present study does not assess feasibility, cost-effectiveness, or system-level impact. Instead, it provides empirically grounded insights into user expectations and acceptance conditions that may inform future implementation research and policy discussions.

### 4.5. Hypothetical Nature of the Concept

It is essential to emphasize that the AI-based care companion discussed in this study represents a hypothetical construct derived from an exploratory needs assessment. No existing application was evaluated, and no evidence regarding effectiveness, safety, or sustained use is provided.

Accordingly, the findings should be interpreted as contributing to early-stage concept development rather than intervention evaluation. They outline perceived needs, desired functions, and acceptance criteria from the perspective of family caregivers. Future research should build on these insights through iterative prototype development, usability testing, effectiveness trials, and ethical evaluation. In particular, studies incorporating objective measures of physical workload, longitudinal designs, and diverse caregiver populations are needed to assess whether AI-based support can meaningfully complement existing care structures without exacerbating inequality.

## 5. Conclusions

This exploratory study provides insight into perceived stress patterns, unmet support needs, and attitudes toward a hypothetical AI-based care companion among family caregivers in Germany. Given the small, heterogeneous, and non-representative sample, the findings should be interpreted as descriptive and indicative rather than as generalizable evidence for the broader family caregiver population. Within these limitations, the results indicate a high subjective burden, with psychological strain emerging as the most frequently reported dominant stress domain. Difficulties in accessing psychosocial support were common, reflecting persistent structural and personal barriers documented in previous research.

Beyond descriptive findings, the exploratory quantitative analyses suggest a strong association between perceived family caregiver burden and openness toward AI-based support. Higher levels of subjective stress were accompanied by greater willingness to use a digital care companion, indicating situational receptiveness to low-threshold, technology-based assistance under conditions of increased strain. Observed group differences related to demographic and caregiving characteristics further underscore the relevance of contextual and life-course factors in shaping acceptance of digital support solutions.

Importantly, the AI-based care companion discussed in this study represents a hypothetical concept derived from family caregivers’ expressed needs and expectations rather than an evaluated intervention. The findings, therefore, do not demonstrate effectiveness but delineate acceptance conditions, desired functions, and perceived risks from the user perspective. Central requirements include non-judgmental communication, personalization, professional reliability, robust data protection, and transparent integration with human support structures.

From a practical perspective, the results suggest differentiated implications for key stakeholders. For policymakers, the findings highlight the need to consider family caregivers explicitly in digital health strategies and to support low-threshold, publicly accessible solutions that do not exacerbate existing inequalities. For developers, the results emphasize the importance of participatory, user-centered design, with a focus on personalization, ethical safeguards, and clear system boundaries. For researchers, the study underscores the value of early-stage needs assessments while also pointing to the necessity of rigorous evaluation designs.

Taken together, the findings suggest that AI-based care companions could potentially complement existing support services by addressing gaps in emotional support, information provision, and early overload detection. However, responsible implementation requires careful ethical consideration, transparent system design, and embedding within established counseling and care infrastructures.

Future research should extend this exploratory work by incorporating objective measures of physical workload, fatigue, and movement-related strain to capture the multidimensional nature of family caregiver burden. In addition, subsequent studies should progress toward the development and iterative testing of concrete prototypes, including usability studies, effectiveness trials, and evaluations across specific family caregiver subgroups (e.g., high-intensity family caregivers, older family caregivers, or those with limited digital literacy). Scaling approaches and implementation pathways within existing care systems should also be systematically examined to assess whether AI-based care companions can meaningfully contribute to sustainable, user-centered support for family caregivers.

## Figures and Tables

**Table 1:** Demographic data of participants.

Category	Answers	Number	Percentage
Gender	Female	45	81.8
	Male	10	18.2
Age	Under 30 years old	10	18.2
	30–39 years old	20	36.4
	40–49 years old	10	18.2
	50–59 years old	13	23.6
	60–69 years old	2	3.6
Highest level of education	University degree	9	16.4
	Other	1	1.8
	Secondary school diploma	18	32.7
	Vocational training	24	43.6
	High school diploma	3	5.5
Care period	Less than 6 months	6	10.9
	6–12 months	8	14.5
	1–5 years	25	45.5
	More than 5 years	16	29.1
Hours of care per week	Less than 10 h	16	29.1
	10–20 h	18	32.7
	21–30 h	6	10.9
	More than 30 h	15	27.3

## Data Availability

The raw data supporting the conclusions of this article will be made available by the authors upon request.

## Supplementary Materials

The following supporting information can be downloaded at https://www.mdpi.com/article/10.3390/healthcare14050586/s1, Overview of the questionnaire items.
