# Supplementary material for: Digital Support for Family Caregivers: Potential and Challenges of a Hypothetical AI Care Companion
Source: Healthcare (Basel). 2026 Feb 26;14(5):586. doi: 10.3390/healthcare14050586 (PMC12984850; doi:10.3390/healthcare14050586)
Supplement: Supplementary file 1 [file healthcare-14-00586-s001.zip › healthcare-4121627-supplementary.pdf]

Overview of the questionnaire items

1. How much stress do you feel overall as a result of your caregiving activities?
- Very strong
- Strong
- Moderate
- Weak
- Not at all

2. Which area is most stressful for you?
- Mental strain (stress, worries, excessive demands)
- Physical strain
- Social isolation

3. Have you ever had difficulty obtaining psychological or psychosocial support?
- Yes
- No
- Not attempted

4. If so, what obstacles did you encounter?
- Long waiting times
- Costs/financing
- Shame/inhibition threshold

5. Could you imagine using a digital AI care companion?
- Yes, definitely
- Yes, probably
- Maybe / I don't know
- Unlikely
- No

6. Which feature would be particularly helpful for you?
- Emotional support / offer of conversation
- Early warning system in case of personal overload
- Information and advice function (care knowledge, tips)
- Reminders of tasks, medication management
- Crisis support / emergency information
- Individual recommendations for relief

7. How important is it to you that the AI companion communicates in a non-judgmental way?

- Very important
- Important
- Neutral
- Somewhat unimportant
- Unimportant

8. Which device would you prefer to use the AI companion on?
- Smartphone
- Computer / Laptop
- Tablet
- Phone / Voice Assistant

9. What concerns would you have about an AI care assistant?
- Data protection/security
- Incorrect advice
- Distrust of AI
- Not human enough
- No concerns

10. How important would it be to you to personalize the AI companion using a questionnaire (biomarkers, stress profile)?
- Very important
- Important
- Neutral
- Somewhat unimportant
- Unimportant

11. How often would you likely use such an AI companion?
- Daily
- Several times a week
- Weekly
- Rarely
- Never

12. Was müsste ein KI-Pflegebegleiter leisten, damit er für Sie wirklich hilfreich wäre? (Open question)

13. How old are you?
- Under 30 years old
- 30–39 years old
- 40–49 years old
- 50–59 years old

- 60–69 years old
- 70 years old and older

14. What is your gender?
- Male
- Female
- Diverse
- No preference

15. What is your highest level of education?
- No degree
- Secondary school diploma
- High school diploma
- A-levels
- Vocational training
- University degree
- Other

16. How long have you been caring for a relative in need of care?
- Less than 6 months
- 6-12 months
- 1-5 years
- More than 5 years

17. How many hours per week do you spend on average providing care?
- Less than 10 hours
- 10-20 hours
- 21-30 hours
- More than 30 hours
